# 

**Published:** 1923-05

**Authors:** 


### PUBLIC HEALTH IN PARLIAMENT.

The following answers have been given in the House of Commons by the Minister of Health.

### HOUSING.

State Assisted Houses.—The difference between the number of State-assisted houses completed up to February 1 last and the gross estimate of shortage due to overcrowding according to the survey referred to is 312,505. Information is not available as to the number of unsubsidised houses built since 1919.

Before and After the War.—Statistics handed to the President of the Local Government Board in September, 1916, by a deputation from the National Congress on "Home Problems after the War" convened by the National Housing and Town Planning Council showed that in the years 1906-1910 the total number of houses built in 70 towns was 169,996, while in the period from 1911-1915 the number was 87,654.

Structurally Separate Dwellings.—According to the Census of 1921, it appears that the number of structurally separate dwellings increased by 357,000 in England and Wales between 1911 and 1921.

Local Authorities.—A number of local authorities are proceeding with the erection of houses in anticipation of the new Housing Bill, and the fourth sub-clause of clause 1 of the Bill provides that local authorities will not be prejudiced as regards the subsidy on account of their having so acted. Any loss on such schemes beyond the amount of the Government subsidy will, of course, fall on the local rates.

Reduction in Tenders.—Tenders recently approved show a fall of approximately 30 per cent. from the prices tendered in the early part of last year, and there is no reason to suppose that this reduction of tender prices differs materially from that which has taken place concurrently in the cost of building. There are not at present any indications of attempts to force up building prices.

Bricklayers' Output.—Accounts as to the number of bricks laid per day in ordinary housebuilding and as to the output of building trade operatives as compared with 1913 vary so much that I am unable to offer any reliable expression of opinion. I will, however, enquire further into this matter. The supply of labour at present available would probably be insufficient to carry out a very considerable housing programme. It will, however, I hope, be found practicable to increase it gradually by natural methods as the demand for it rises.

### PUBLIC HEALTH.

Cancer Death-rate.—The standard death-rates from cancer in England and Wales in the four decades 1881-1890, 1891-1900, 1901-1910, and 1911-1920, and in the year 1921 were 610, 767; 867, 966 and 1,007 per million of the population respectively. In England and Wales the cancer deaths during the above decades represented 3.2, 4.3, 6.1, and 8.0 per cent. of the total deaths from all causes, other than those due to accident and violence.

Mentally Affected Ex-Service Men.—On January 1 last the number of ex-service men in mental hospitals in England and Wales classed as service patients and paid for by the Ministry of Pensions was 4,532.

### NATIONAL HEALTH INSURANCE.

Cost of Administration.—The total costs of administration (central and local) in the two cases are at present: National Health Insurance, 6s. 8d. per insured person, and Unemployment Insurance 7s. 1d.

Panel Doctors (Remuneration).—Before the expiry of the present agreement at the end of this year, both the terms of service and the remuneration of insurance practitioners will have to be reconsidered. The Insurance Consultative Council have already been consulted in regard to the terms of service and will be consulted also as to the rate of remuneration. There is no other body representative of all the approved societies, but I shall be prepared to receive representations from the principal groups before coming to a final decision.

Insurance Committee's Decisions.—I have no jurisdiction to review an Insurance Committee's decisions unless the doctors concerned exercise their right of appeal to me. There must be some safeguard against extravagance in prescribing, but the present arrangements are not working well and are under revision.

### TUBERCULOSIS.

Spahlinger Treatment.—All the evidence available as to the value of this treatment for tuberculosis has been investigated by my Department, and one of the medical officers of the Ministry visited Geneva last year, and, through the courtesy of M. Spahlinger, was enabled to examine certain cases of tuberculosis which have been treated by this method. The conclusions arrived at were that, although it is not yet possible to express an opinion upon the scientific value of M. Spahlinger's work from the bacteriological standpoint, the clinical results already obtained fully warrant further investigation as soon as a supply of the complete serum and vaccine is available. It is understood that no definite date can yet be fixed by M. Spahlinger for the production of a further supply even for the purposes of scientific investigation. I am most anxious to encourage further trial of the remedies in this country under expert observation as soon as a supply is available.

Tuberculosis by Telephone.—As the result of bacteriological inquiries made in this country and abroad some few years ago, it was held that the transmission of pulmonary tuberculosis through the medium of the telephone mouthpiece is practically impossible. In any event, the disinfection of telephone mouthpieces after each individual user would obviously be impracticable.

Total Number of Deaths.—The total number of deaths in England and Wales from all forms of tuberculosis in 1922 was 42,777, or an average of 117 per day.

### RECENT APPOINTMENTS.

### Medical Officers, Chaplains, etc.

Adamson, Alfred F., M.B., Ch.B.; Superintendent, East Pilton Sanatorium, Edinburgh; Assistant M.O.H., West Hartlepool.

Bates, Hubert Tunstall; M.O.H. to Ilkley Urban Council.

Blackburn, W. Howard, M.R.C.S., L.R.C.P.; Assistant M.O.H., St. Helens.

Joyner, Charles, M.A., M.B., Ch.B.: Medical Officer, King·

Gowers, D. W., Assistant Medical Officer, East Ham.

seal Mental Hospital, Aberdeen; Junior Assistant M.O., County Hospital, Mickleover, Derby.

Morris, John Mudie; Medical Officer of Health, Neath, £750.

Shennan, A. H., Assistant Tuberculosis Officer, Edinburgh Corporation; Assistant Tuberculosis Officer, and Assistant School M.O., Lindsey County Council (Edinburgh Academy).

Higgins, Stadick, M.D., F.R.C.S., L.R.C.P., M.O.H. St Pancras; M.O.H. Capetown.

Maclean, Surgeon Rear-Admiral Alexander, D.S.O.; M.O. Haslar Hospital.

### Public Health and Hospital Appointments.

Bailey, C. E., Matron of the North Riding Infirmary, Middlesbrough; Matron, General Hospital, Birmingham.

Davies, Major H. Tudor, M.C.; Chief Engineering Assistant, Cardiff County Council.

Ellis, Walter; Sanitary Inspector, Hemsworth Urban District Council.

Fisher, Albert; Senior Sanitary Inspector, Colchester.

Tobitt, C. R.; Sanitary Inspector and Surveyor, Halstead Rural District Council.

Joy, Eustace, Clerk to the Staffordshire County Council; Clerk to the Staffordshire Mental Hospitals Board.

At a meeting of the Governors of the City of London Hospital for Diseases of the Chest recently held, it was decided to alter the name of the Institution to "The City of London Hospital for Diseases of the Heart and Lungs," which title now more completely describes the character of its present work.

